# Dose–Response Effects of 3-Nitrooxypropanol Combined with Low- and High-Concentrate Feed Proportions in the Dairy Cow Ration on Fermentation Parameters in a Rumen Simulation Technique

**DOI:** 10.3390/ani11061784

**Published:** 2021-06-15

**Authors:** Matthias Schilde, Dirk von Soosten, Liane Hüther, Susanne Kersten, Ulrich Meyer, Annette Zeyner, Sven Dänicke

**Affiliations:** 1 Institute of Animal Nutrition, Friedrich-Loeffler-Institut (FLI), Federal Research Institute for Animal Health, 38116 Braunschweig, Germany; Dirk.von_Soosten@fli.de (D.v.S.); Liane.Huether@fli.de (L.H.); Susanne.Kersten@fli.de (S.K.); Ulrich.Meyer@fli.de (U.M.); Sven.Daenicke@fli.de (S.D.); 2 Institute of Agricultural and Nutritional Sciences, Group Animal Nutrition, Martin Luther University Halle-Wittenberg, 06120 Halle (Saale), Germany; annette.zeyner@landw.uni-halle.de

**Keywords:** 3-nitrooxypropanol, concentrate feed proportion, RUSITEC, methane inhibitor, methane production

## Abstract

**Simple Summary:**

Feeding strategies which aim at mitigating ruminal methane formation, a significant contributor to total greenhouse gas emissions, are being continuously developed, yet they need to be investigated in relation to their effectiveness and the mechanisms behind their effects in vitro before they undergo further assessment in vivo. In this context, the present study investigated the dose–response relationships of the methane inhibitor 3-nitrooxypropanol supplemented to varying concentrate feed proportions in a rumen simulation technique. Methane production was effectively reduced with an increasing dose of 3-nitrooxypropanol, which was, however, independent of concentrate feed proportion. Total gas production and fibre degradability were not affected by 3-nitrooxypropanol, indicating no negative side effects on fermentative capability. However, the hydrogen-liberating acetate production was reduced, whilst hydrogen gas was notably increased in a dose-dependent manner. The present in vitro study provides a deeper insight into a combined (3-nitrooxypropanol and high-concentrate feed proportions) methane abatement strategy under controlled conditions. The present combined approach reveals neither negative side effects nor additive effects between 3-nitrooxypropanol and varying concentrate feed proportions, which should be further investigated in future experiments in vivo.

**Abstract:**

Methane (CH_4_) from ruminal feed degradation is a major pollutant from ruminant livestock, which calls for mitigation strategies. The purpose of the present 4 × 2 factorial arrangement was to investigate the dose–response relationships between four doses of the CH_4_ inhibitor 3-nitrooxypropanol (3-NOP) and potential synergistic effects with low (LC) or high (HC) concentrate feed proportions (CFP) on CH_4_ reduction as both mitigation approaches differ in their mode of action (direct 3-NOP vs. indirect CFP effects). Diet substrates and 3-NOP were incubated in a rumen simulation technique to measure the concentration and production of volatile fatty acids (VFA), fermentation gases as well as substrate disappearance. Negative side effects on fermentation regarding total VFA and gas production as well as nutrient degradability were observed for neither CFP nor 3-NOP. CH_4_ production decreased from 10% up to 97% in a dose-dependent manner with increasing 3-NOP inclusion rate (dose: *p* < 0.001) but irrespective of CFP (CFP × dose: *p* = 0.094). Hydrogen gas accumulated correspondingly with increased 3-NOP dose (dose: *p* < 0.001). In vitro pH (*p* = 0.019) and redox potential (*p* = 0.066) varied by CFP, whereas the latter fluctuated with 3-NOP dose (*p* = 0.01). Acetate and *iso*-butyrate (mol %) decreased with 3-NOP dose, whereas *iso*-valerate increased (dose: *p* < 0.001). Propionate and valerate varied inconsistently due to 3-NOP supplementation. The feed additive 3-NOP was proven to be a dose-dependent yet effective CH_4_ inhibitor under conditions in vitro. The observed lack of additivity of increased CFP on the CH_4_ inhibition potential of 3-NOP needs to be verified in future research testing further diet types both in vitro and in vivo.

## 1. Introduction

Methane (CH_4_) is a climate-relevant greenhouse gas with a direct environmental impact insofar as its global warming potential exceeds 28 times that of carbon dioxide (CO_2_) on a 100-year time horizon [1]. In particular, enteric CH_4_ from feed fermentation contributes to 46% of the total emissions from the dairy supply chain worldwide [2]. Accordingly, the development and implementation of CH_4_ abatement strategies in ruminant livestock production systems can be expected to gain in importance [3].

The rumen simulation technique (RUSITEC) was introduced by Czerkawski and Breckenridge [4] as a semi-continuous-flow system to facilitate investigations on rumen fermentation processes, such as CH_4_ production, and its manipulation under strictly controlled conditions. In parallel, dose–response relationships can be examined in the RUSITEC by incubating different dosage levels of CH_4_ inhibitor substances on diet substrates in the juxtaposed reaction vessels.

Methane formation in ruminants, being catalysed by methyl Coenzyme M reductase (MCR) in hydrogenotrophic methanogenic Archaea, is the major pathway of removing metabolic hydrogen by reduction of CO_2_ [5]. Apart from intraruminal volatile fatty acid (VFA) synthesis, CO_2_ and hydrogen (H_2_) result from microbial degradation of fibre as well as non-fibre carbohydrates (NFC) supplied by the feed ration. 

The synthetic substance 3-nitrooxypropanol (3-NOP) is a direct CH_4_ inhibitor and structural analogue of methyl-coenzyme M (CoM). Thus, 3-NOP binds to the active site of the nickel enzyme methyl-coenzyme M reductase (MCR), causing its inactivation by oxidising the Ni(I) to Ni(II) in the cofactor F_430_. As a consequence, the MCR catalysed the reduction of CoM with coenzyme B to CH_4_ and the heterodisulphide is intermitted during the last step of methanogenesis [5]. In contrast, increasing concentrate feed proportions (CFP) in the feed ration were previously proven as an indirect CH_4_ abatement strategy [6], which can be related to diet-dependent effects on microbial community structures [7], reduced rumen pH values being detrimental to the growth of pH-sensitive methanogens and fibrolytic bacteria [8], and alterations in fermentation pathways. Thus, higher contents of NFC in high-concentrate diets are mainly degraded by propionate enhancers and, therefore, redirected to H_2_-consuming fermentation pathways, which results in substrate competition with methanogenesis [9]. 

Significant dose–response relationships of 3-NOP on CH_4_ mitigation were observed in vitro [10] and in vivo [11,12,13]. Romero-Pérez et al. [10] tested 500, 1000, and 2000 mg of 3-NOP/kg of feed DM incubated with a high-forage diet substrate in a RUSITEC and observed quadratic effects of 3-NOP dose on CH_4_ reduction (76.0%, 84.5%, and 85.6%). However, little information has been reported to reveal the dose–response relationships of 3-NOP in consideration of the potential additive effects with low and high CFP. Romero-Pérez et al. [14,15] supplemented 3-NOP in combination with the ionophore monensin to either high-forage [14] or high-grain [15] diets in a RUSITEC and reported additive effects of neither monensin nor high-grain diets on CH_4_ reduction. 

Regarding in vivo experiments, Vyas et al. [13] supplemented 50, 75, 100, 150, and 200 mg of 3-NOP/kg of feed dry matter (DM) to beef cattle provided high-forage and high-grain diets. The authors observed a significant dose response with regard to the higher dosage levels of 100, 150, and 200 mg of 3-NOP and a significant effect of the ration type. Thus, 3-NOP efficacy was greater in high-grain (26, 33, and 45% CH_4_ reduction, resp.) when compared to high-forage (16, 21, and 23% CH_4_ reduction, resp.) diets. However, CH_4_ emissions at 50 and 75 mg 3-NOP dose/kg feed DM were not significantly different from the control. Correspondingly, in a meta-analysis of 3-NOP experiments including dairy and beef cattle, Dijkstra et al. [11] confirmed the dose-dependent 3-NOP effect on CH_4_ yield, which was modelled to −2.48 ± 0.0734% CH_4_ yield per 10 mg/kg DM increase in 3-NOP dose from its mean (123 mg 3-NOP/kg of feed DM). Melgar et al. [12] mixed 3-NOP into a forage-based total-mixed ration (TMR) for dairy cows and reported that CH_4_ yield quadratically decreased by 24.3, 26.5, 22.5, 33.5, 35.9, and 31.8% for 40, 60, 80, 100, 150, and 200 mg of 3-NOP/kg feed DM, respectively, with no statistical difference among 40, 60, and 80 as well as between 100, 150, and 200 mg 3-NOP/kg DM. 

Accordingly, in vitro studies investigating the dose–response relationships of 3-NOP in combination with low- and high-concentrate diets are scarce. Therefore, the present RUSITEC experiment aimed at investigating the dose–response relationships of 3-NOP and potential synergistic effects between 3-NOP dosage level and low- or high-concentrate diets on fermentation parameters. A novelty of the present approach encompasses the application of very low 3-NOP inclusion rates, which were experimentally chosen to enable comparisons to those recently supplemented to dairy [12,16] and beef [13] cattle (40–200 mg 3-NOP/kg feed DM) under practical conditions in vivo. 

It was hypothesised that CH_4_ production decreases with increasing 3-NOP dosage level and that supplementing high-concentrate feed proportions causes additive effects on CH_4_ reduction in vitro.

## 2. Materials and Methods

The experiment was carried out at the experimental station and laboratory of the Friedrich-Loeffler Institut (FLI) in Braunschweig, Germany. Maintenance of the cannulated cows and collection of rumen fluid were in compliance with the German Animal Welfare Act and approved by the Lower Saxony State Office for Consumer Protection and Food Safety (LAVES), Germany (33.19-42502-04-15/1858). 

### 2.1. Experimental Design and Diets

The experiment was conducted using the RUSITEC according to the general incubation procedure described by Czerkawski and Breckenridge [4]. The study was arranged as a 2 × 4 factorial design with low- (LC) and high- (HC) concentrate feed proportion (CFP) in the incubated diet and the methane inhibitor 3-nitrooxypropanol (3-NOP; DSM Nutritional Products AG, Kaiseraugst, Switzerland) supplied at four doses of 0 (placebo, PLA), 73 (LOW), 160 (MED), and 1200 (HIGH) mg of the active 3-NOP substance/kg of feed DM. Both the placebo and the 3-NOP supplement contained propylene glycol and SiO_2_ acting as carriers for 10% of the active 3-NOP substance (1,3-propanediol mononitrate) in product DM, which was included in 3-NOP treatments only. On a DM basis, the experimental diet substrates were formulated according to the forage:concentrate ratio of 70:30 (LC) and 40:60 (HC). The forage proportion of the LC and HC diet was composed of 70% maize silage and 30% grass silage. The 3-NOP supplement was mixed into the ground concentrate feed and homogenised for 10 min (M4.REI; Gebr. Lödige Maschinenbau GmbH, Paderborn, Germany). 

### 2.2. Experimental Procedure

In total, six incubation trials were conducted using a four-vessel RUSITEC apparatus. Each CFP × 3-NOP combination was tested in triplicate. Each incubation run consisted of an adaptation period lasting eight days, followed by a four-day sampling period.

The diet components were pre-dried at 60 °C for 48 h and ground to pass a 10 mm (forages) and a 3 mm (concentrates) screen (SM 1, Retsch, Haan, Germany). The diet (12.0 g fresh matter (FM) with 90.3% DM content) was weighed into nylon bags (50 ± 15 μm pore size; 10 × 20 cm; ANKOM Technol., Fairport, NY, USA). The ingredients and chemical composition of the incubated feedstuffs and diets are presented in Table 1.

Three rumen-fistulated cows were kept as donor animals for the inoculum of rumen liquid and solid digesta on a diet consisting of 40% concentrates, 30% maize silage, and 30% grass silage (DM basis) for ad libitum intake. Inocula were collected from three cows via the fistula one hour before the morning feeding. Rumen fluid was collected by introducing a probe [17], which was attached to the flexible tube of a hand suction pump (SELEKT Rumen-Fluid Collector, Nimrod Veterinary Products Ltd., Gloucestershire, UK), into the ventral rumen. Solid rumen digesta were manually taken from the ventral, caudal, and cranial side of the rumen. The fluid was strained (cheesecloth of 250 µm mesh opening) into nitrogen-flushed and pre-warmed insulated bottles. Both the solid and liquid rumen contents were placed into in a water bath (39 °C), transported to the laboratory immediately, and pooled together.

The incubation was initiated by inoculating the pre-warmed reaction vessels (volume of 900 mL), each with 550 mL of rumen fluid, 100 mL of warm artificial saliva [18], and with one nylon bag of 80 g wet weight of solid rumen digesta and one nylon bag containing the diet substrate. Subsequently, the bags were inserted into the perforated feed container of each vessel and the fermenters were immersed in the water bath (39 °C) of the RUSITEC apparatus. The food containers were moved up and down (vertical strokes of 65 mm) and agitated at 8 cycles/min. The feed bags were incubated for 48 h in the food container, whereas the initial bag containing the solid rumen inoculum was replaced after 24 h by a feed bag. After 48 h of incubation, the feed bags were removed from the vessel, gently washed with 40 mL of artificial saliva for 1 min in polyethylene bags, squeezed by hand, and replaced by a new one. The washed-out fluids were returned into the vessel. Both the vessels and effluent bottles were flushed with nitrogen every day after feed bag exchange. The McDougall buffer solution [18] was prepared daily and similarly across treatments and continuously infused into each vessel to achieve a dilution rate of 650 mL/24 h (3%/h) using a peristaltic pump. Buffer composition and infusion rate were not changed between treatments to investigate inherent effects of the diet composition in combination with 3-NOP.

### 2.3. Sampling and Analyses

Feed samples of the pre-incubated and fermented diet were analysed according to the standard methods of the Association of German Agricultural Analytic and Research Institutes [19] for DM (3.1), crude ash (CA; 8.1), crude protein (CP; Dumas method, 4.1.2), ether extract (EE) pre-treated with hydrochloric acid (5.1.1), starch (7.2.1), acid detergent fibre (ADFom; 6.5.2), and α-amylase treated neutral detergent fibre (aNDFom; 6.5.1), both expressed without residual ash. 

During the four-day sampling period, all the samples were taken contemporaneously for one daily feed bag exchange.

Each feed bag collected after 48 h incubation was dried at 60 °C for 72 h, weighed, and ground to pass a 1-mm mesh sieve (SM 1; Retsch, Haan, Germany). The feed residues of each treatment and incubation run were pooled over the sampling period and analysed for DM, CA, and aNDFom.

The pH and redox potential (*Eh*) in the fermenter fluid were measured using glass electrodes (SenTix 41 (pH) and SenTix PtR (*Eh*); pH 7110; WTW, Weilheim, Germany) which were calibrated every day.

The effluent was collected in 1-litre volumetric flasks placed on ice and effluent volume was noted daily. VFA were analysed from daily collected effluent samples (80 mL) according to Geissler et al. [20] using a gas chromatograph (Clarus 680; PerkinElmer LAS GmbH, Rodgau, Germany) equipped with a flame ionisation detector. Ammonia-N concentration (NH_3_-N) was measured using steam distillation (DIN38406-E5-2, [21]).

Fermentation gases were collected over the whole sampling period in 10-litre gas bags (Plastigas; Linde GmbH, Pullach, Germany). After termination of the run, 10 mL of fermentation gases were withdrawn via the septum of the gas bag using a gas-tight syringe. The gas samples were injected on a chromatography column (Porapak QS; 80/100 mesh, 3 m × 3 mm, Agilent Technologies, Inc., Santa Clara, CA, USA) of a gas chromatograph equipped with a thermal conductivity detector (GC-14B; Shimadzu, Kyoto, Japan) and argon as carrier gas. Gas samples were determined for percentage of CH_4_, CO_2_, and H_2_. The gas volume in the gas bags was measured using a drum-type gas meter (TG05; Ritter Apparatebau GmbH & Co. KG, Bochum, Germany) and added to the gas volume of the gas space in the effluent bottle. 

### 2.4. Calculations and Statistical Analyses

Total gas volume was corrected for temperature (0 °C) and pressure (101.325 kPa) conditions. The daily production of VFA and NH_3_-N resulted from multiplication of the measured concentrations by the effluent and gas volume, respectively. Apparent disappearance of organic matter (OMAD) and the degradability of DM (DMD) and aNDFom (NDFD) after 48 h of incubation were calculated by subtracting the pre- and post-incubated nutrient contents and substrate masses.

Statistical data analysis was carried out using PROC MIXED (version 9.4; SAS Institute Inc., Cary, NC, USA) and the mixed model was fitted by a restricted maximum likelihood (REML) method according to Littell et al. [22]. The 3-NOP dose level (DOSE), concentrate proportion in the diet substrate (CFP), incubation run, and their interaction were set as fixed effects and fermentation vessel was implemented as a random effect. Satterthwaite approximation was used for calculating the degrees of freedom. The variance components were estimated using the REML method and the variance–covariance structure was selected based on the lowest Akaike Information Criterion. Customised post-fitting hypothesis tests among LS means were conducted using the LSMESTIMATE statement in PROC MIXED with SCHEFFE-adjusted multiple comparisons.

To fit the nested polynomial regression model and convert 3-NOP doses to equally spaced dosage levels, the linear LOGDOSE = LOG2(DOSE) and quadratic LOGDOSE_2 = LOGDOSE^2 regression parameters were created for 3-NOP doses within each CFP in the DATA step. As fixed regressive components were considered the effects of CFP, increasing 3-NOP dose (LOGDOSE) within the treatment (LC or HC) “LOGDOSE(CFP)” (linear regression term) and, for calculating the quadratic regression term, additionally its square “LOGDOSE_2 (CFP)”. RUN × DOSE was set in the RANDOM statement to define the whole-plot error. The HTYPE option was set = 1 to enter and test the model terms (linear, quadratic) in sequential order.

The CONTRAST statement was used to test whether regression coefficients (linear (L), quadratic (Q)) were equal between both treatments of CFP (*H*_0_: *ß_L,HC_* = *ß_L,LC_* and *H*_0_: *ß_Q,HC_* = *ß_Q,LC_*). The t-values from the regression model were used to test linear and quadratic effects of 3-NOP: *H*_0_: *ß_L,3-NOP_* or *ß_Q,3-NOP_* = 0, which is equivalent to the orthogonal polynomial contrasts.

PROCEDURE MIXED METHOD = REML;

CLASS CFP RUN DOSE;

MODEL Y = CFP LOGDOSE(CFP) LOGDOSE_2(CFP)/NOINT DDFM = KENWARDROGER SOLUTION HTYPE = 1;

RANDOM RUN RUN × DOSE;

CONTRAST ‘LINEAR: coefficients equal’ LOGDOSE(CFP) 1 −1;

CONTRAST ‘QUADRATIC: coefficients equal’ LOGDOSE _2 (CFP) 1 −1;

RUN.

Effects were declared statistically significant at *p*-values ≤ 0.05 and a trend was postulated at *p*-values between >0.05 and 0.10. Results are presented as least square means (LS means) with the standard error of means (SEM). Pearson correlation coefficients were calculated with N = 24 observations.

## 3. Results

### 3.1. Diet Composition and Substrate Degradability

Ingredients and chemical composition of the incubated diets are presented in Table 1.

The DMD (dose; *p* = 0.041) and OMAD (dose: *p* = 0.052) varied by 3-NOP dosage level (Table 2). In LC diets, DMD and OMAD increased from PLA to LOW by 11% and decreased by 7% in HIGH when compared to PLA. The DMD and OMAD were comparable between PLA and MED. In HC, DMD and OMAD were highest in diets with LOW and HIGH 3-NOP dosage levels but lower in PLA and MED. Percentage of DMD (%) was positively related to percentage proportion of CH_4_ (Vol.-%) (*r* = 0.471; *p* = 0.020) and CO_2_ (Vol.-%) (*r* = 0.487; *p* = 0.016) but negatively to H_2_ (Vol.-%) (*r* = −0.368; *p* = 0.077) in total fermentation gas.

Degradability of NDF tended to be higher in LC diets (CFP: *p* = 0.091; Table 2) irrespective of 3-NOP dose. The NDFD correlated negatively with pH in fermenter fluid (*r* = −0.665; *p* < 0.001) and NH_3_-N (mg/g of DMD) (*r* = −0.632; *p* = 0.001) but positively to *Eh* (*r* = 0.495; *p* = 0.014) and acetic acid concentration (mmol/L) (*r* = 0.734; *p* < 0.001).

### 3.2. Gas Production and Gas Composition

Total GP (mL/d and mL/g of DMD) and CO_2_ (% and mL/g of DMD) were affected by neither 3-NOP dose nor CFP (Table 3) and both were negatively correlated to pH in the fermenter fluid (GP: *r* = −0.436; *p* = 0.033; CO_2_: *r* = −0.460; *p* = 0.024). A trend was observed for a quadratic effect of 3-NOP dose on CO_2_ (Vol.-%) regarding LC diets (Q: *p* = 0.066; Table 3) and the difference in quadratic regression coefficients between LC and HC diets (*ß*_Q_ > *F*: *p* = 0.086). 

The greatest CH_4_ proportion (5.8%) was recorded for the control (PLA), followed in descending order by LOW, MED, and HIGH 3-NOP treatment down to 0.2% as in the case referring to CH_4_ production (from 15.8 to 0.5 mL/g of DMD) in LC diets and 5.5% (PLA) to 0.5% (HIGH) and 14.5 (PLA) to 1.1 mL/g of DMD (HIGH) in HC diets, respectively (Table 3; dose: *p* < 0.001). Increasing 3-NOP dosage levels reduced CH_4_ (% and mL/g of DMD) in a linear manner in LC diets only (L: *p* < 0.01; Table 3) and, with regard to CH_4_ (Vol.-%), 3-NOP efficacy tended to be less pronounced in HC substrates (CFP × dose: *p* = 0.094; Table 3). The linear (*ß*_L_ > *F*: *p* = 0.028) and quadratic (*ß*_Q_ > *F*: *p* = 0.045) components of the regression were significantly different between LC and HC, indicating a variation in 3-NOP mitigation efficiency depending on the provided CFP, whereas the CFP main effect was not significant. In LC diets, 3-NOP supplementation reduced CH_4_ (Vol.-%) by 12% (LOW), 61% (MED), and 97% (HIGH) relative to CH_4_ (Vol.-%) analysed in the fermentation gas of the PLA treatment. Comparatively, CH_4_ (Vol.-%) was mitigated to a lower extent in HC treatments, namely by 10% (LOW), 35% (MED), and 90% (HIGH) in relation to PLA. Methane proportion (Vol.-%) and production (mL/g of DMD) significantly differed among 3-NOP doses, except in the PLA versus LOW treatments (CH_4_ (Vol.-%): LC: *p* = 0.147; HC: *p* = 0.258 and CH_4_ (mL/g of DMD): LC: *p* = 0.045; HC: *p* = 0.166). Positive correlations (*p* < 0.05) were found between CH_4_ (Vol.-%) and *iso*-butyrate (mol %) (*r* = 0.695), acetate (mol %) (*r* = 0.427), and propionate (mol %) (*r* = 0.420), whereas CH_4_ (Vol.-%) was negatively linearly related (*p* < 0.05) to H_2_ (Vol.-%) (*r* = −0.872), *iso*-valerate (mol %) (*r* = −0.796), and total VFA production (mmol/g of DMD) (*r* = −0.450).

**Figure 1:**
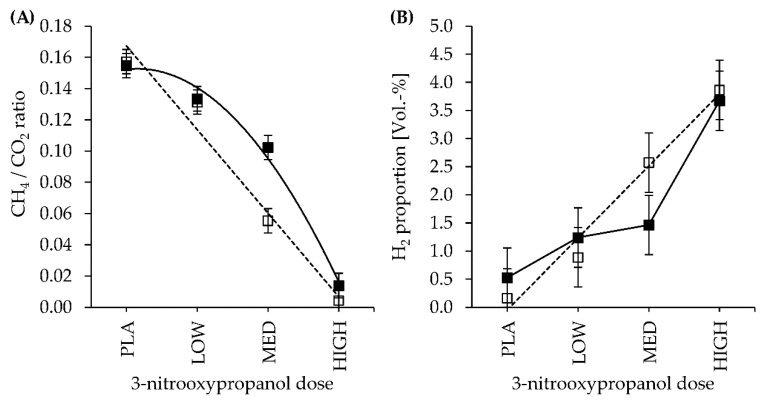
Effect of 3-nitrooxypropanol dose (PLA: 0, LOW: 73, MED: 160, and HIGH: 1200 mg of 3-NOP/kg of feed DM) and low- (□, dashed line) or high- (■, solid line) concentrate proportion in the incubated diet on (**A**) methane (CH_4_) to carbon dioxide (CO_2_) ratio and (**B**) hydrogen (H_2_) proportion (Vol.-%) in fermentation gases; curve fitting according to (non)significant L and Q effects (Table 3).

The ratio of CH_4_/CO_2_ was significantly affected by the CFP × dose interaction (CFP × dose: *p* = 0.026). In HC diets, the CH_4_/CO_2_ ratio tended to be quadratically influenced by 3-NOP dose level (Q: *p* = 0.082), whereas that of LC substrates was affected in a linear dose-dependent manner (L: *p* < 0.001) (Table 3; Figure 1A). The CFP treatment caused significantly different courses of the 3-NOP dose-related CH_4_/CO_2_ ratio as the linear and quadratic regression coefficients significantly differed (*ß*_L_ > *F*: *p* = 0.035; *ß*_Q_ > *F*: *p* = 0.048). Regarding 3-NOP dose MED, CH_4_ was mitigated more effectively in LC compared to HC diets (CH_4_ (Vol.-%) and CH_4_/CO_2_ ratio: contrast LC versus HC for dose MED: *p* < 0.01) (Table 3; Figure 1A). In an inverse ratio, the CO_2_/CH_4_ ratio increased with increasing 3-NOP dose (*p* = 0.036; Table 3). A considerably wider CH_4_/H_2_ ratio was found in the PLA treatment, which was most apparent in the LC diet (CFP × dose: *p* = 0.001). Increasing 3-NOP inclusion levels caused a linear and quadratic decrease in the CH_4_/H_2_ ratio for LC diets (L: *p* < 0.001; Q: *p* = 0.001) and the dose–response curves significantly differed by CFP (*ß*_L_ > *F*: *p* = 0.001; *ß*_Q_ > *F*: *p* = 0.002).

Figure 1B illustrates a 27-fold increase in H_2_ (% and mL/g of DMD) in LC and, to a lesser extent, a 6.2-fold increase in HC diets with increasing 3-NOP dose relative to PLA (Table 3; dose: *p* = 0.001). However, H_2_ (% and mL/g of DMD) was not different between PLA and LOW. Both linear and quadratic effects of 3-NOP dose on H_2_ (Vol.-%) remained not significant concerning the HC diet. In contrast, the linear regression coefficient was significant for the LC diet (L: *p* = 0.046), indicating that the slope of the curve increased more steadily when compared to HC (Figure 1B). In HC diets, H_2_ (% and mL/g of DMD) was not significantly higher for 3-NOP dose MED in comparison to PLA, whereas contrast analysis revealed a significant variation between PLA and MED for LC diets (*p* < 0.05). Furthermore, H_2_ (Vol.-%) significantly correlated to CH_4_ (Vol.-%) (*r* = −0.872), acetate (mol %) (*r* = −0.553), propionate (mol %) (*r* = −0.379; *p* = 0.068), and iso-butyrate (mol %) (*r* = −0.570) in a negative manner, whereas positive relationships (*p* < 0.05) were found for *iso*-valerate (mol %) (*r* = 0.601) and the production of butyrate (mmol/g of DMD) (*r* = 0.606).

### 3.3. Fermentation Parameters and End-Products

**Figure 2:**
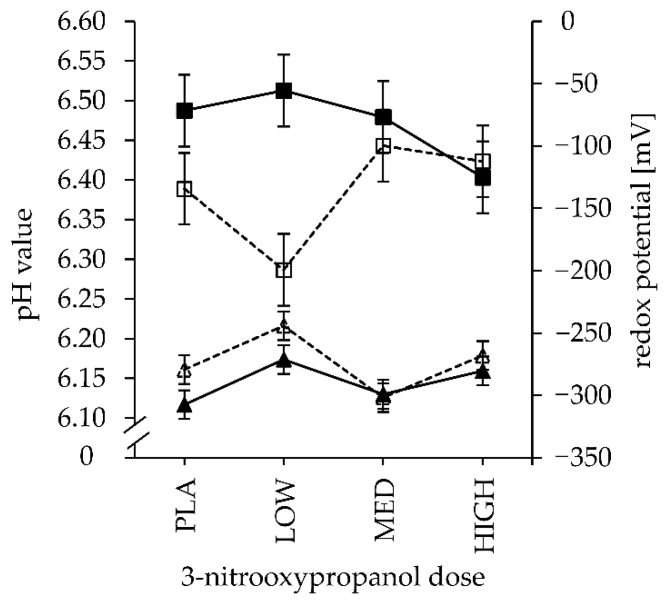
Effect of 3-nitrooxypropanol dose (PLA: 0, LOW: 73, MED: 160, and HIGH: 1200 mg of 3-NOP/kg of feed DM) and low- (□,△, dashed line) or high- (■,▲, solid line) concentrate proportion in the incubated diet on pH values (■,□) and redox potential (▲,△) in fermenter fluid; curve fitting according to (non)significant L and Q effects (see Table 4).

The pH was significantly lower in fermenter fluids of LC diets (CFP: *p* = 0.019; Table 4), which is due to the sharp drop in pH at the MED 3-NOP dose (Figure 2). The *Eh* was affected by 3-NOP dose (dose: *p* = 0.01; Table 4) insofar as the *Eh* of 3-NOP dose LOW was significantly higher when compared to that of the PLA treatment (*p* = 0.008). However, the *Eh* values of MED (*p* = 0.548) and HIGH (*p* = 0.120) were not significantly changed when compared to PLA, but MED differed from 3-NOP doses LOW (*p* = 0.002) and HIGH (*p* = 0.039) (Figure 2).

The effluent volume (mL/d) tended to be interactively affected by CFP and dose (*p* = 0.065) and appeared to be significantly reduced at 3-NOP dose MED and HIGH in relation to LOW in HC diets (Table 4).

Irrespective of 3-NOP dose, NH_3_-N concentration (mg/L) was 41% lower in LC compared with HC diets (CFP: *p* = 0.009; Table 4). In addition, NH_3_-N concentration increased when supplementing 3-NOP dose LOW but decreased at higher 3-NOP doses of MED and HIGH independently of the CFP in the incubated ration (dose: *p* = 0.006).

**Figure 3:**
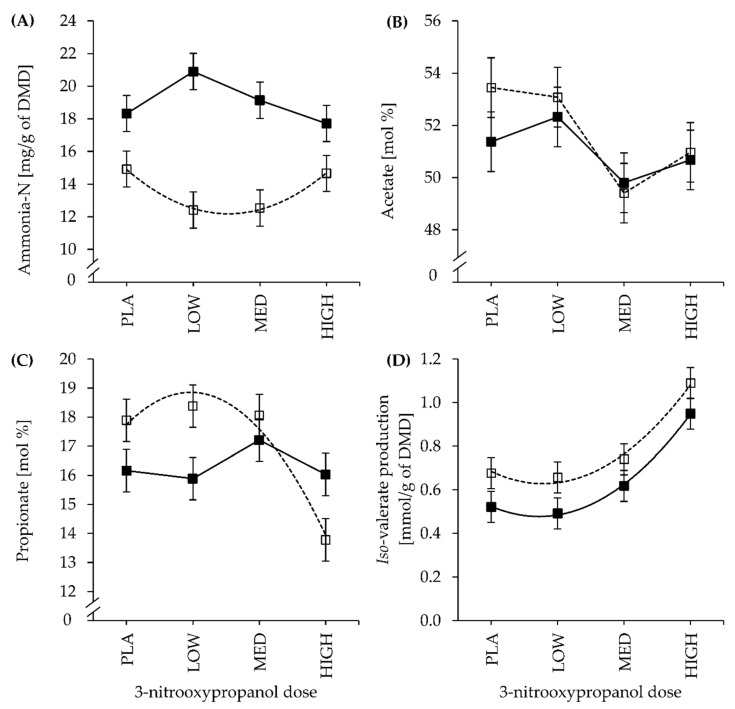
Effect of 3-nitrooxypropanol dose (PLA: 0, LOW: 73, MED: 160, and HIGH: 1200 mg of 3-NOP/kg of feed DM) and low- (□, dashed line) or high- (■, solid line) concentrate proportion in the incubated diet on (**A**) ammonia-N production (mg/g of dry matter degradation (DMD)), (**B**) acetate (mol %), (**C**) propionate (mol %), and (**D**) *iso*-valerate production (mmol/g of DMD) measured in the effluent; curve fitting according to (non)significant L and Q effects (see Table 4).

The NH_3_-N production ranged from 12.4 to 14.9 mg/g of DMD within LC and between 17.7 and 20.9 mg/g of DMD within HC treatment (Table 4). Moreover, NH_3_-N production significantly differed by 5.4 mg/g of DMD between LC and HC (CFP: *p* < 0.001) and tended to be influenced by the CFP × dose interaction (*p* = 0.081). In LC treatments, NH_3_-N production varied in a quadratic manner with increasing 3-NOP dose (Q: *p* = 0.049) (Figure 3A). The curves of NH_3_-N production were shaped in an inverse manner between the incubated LC (convex) and HC (concave) substrates, and the quadratic regression coefficients differed significantly (*ß*_Q_ > *F*: *p* = 0.020).

Total VFA concentration (mmol/L) was not modified by treatments. In contrast, higher total VFA production (mmol/g of DMD) was observed with reduced dietary CFP (CFP: *p* = 0.012) and increasing 3-NOP dose level (dose: *p* = 0.05) (Table 4).

Molar acetate proportion (mol %) ranged from 49.8% to 52.3% in LC and 49.4% to 53.4% in the HC treatment. Acetate (mol %) was not influenced by CFP but decreased with 3-NOP dose increment (dose: *p* = 0.044) and dropped to the greatest extent at 3-NOP dose MED (Figure 3B). In contrast, acetate production (mmol/g of DMD) was independent of 3-NOP dose but 9.4% higher in LC than in HC diets (CFP: *p* = 0.004; Table 4).

The molar percentage of propionate (mol %) and its production (mmol/g of DMD) were numerically increased in LC diets and affected by a CFP × dose interaction (*p* < 0.05), which was related to the notable drop at 3-NOP dose HIGH in the LC treatment when compared to the relatively constant fluctuations observed in the HC treatment. Accordingly, a quadratic effect of 3-NOP dose on propionate (mol %) was noted in LC diets (Q: *p* = 0.017) (Figure 3C).

It was noted that CFP and 3-NOP dose affected the acetate/propionate ratio (C_2_/C_3_) in an interactive manner (CFP × dose: *p* = 0.032). The quadratic effect of 3-NOP dose on C_2_/C_3_ in LC diets (Q: *p* = 0.008) corresponded to the continual decrease from 3.03 to 2.77 in C_2_/C_3_ with increasing 3-NOP dose and the peak of 3.80 at 3-NOP dose HIGH. In HC diets, C_2_/C_3_ fluctuated non-significantly between 3-NOP treatments.

Neither CFP nor 3-NOP dose affected butyric acid (mol % and mmol/g of DMD). Valeric acid (mol % and mmol/g of DMD) was only affected by 3-NOP dose (dose: *p* < 0.05). The steady decrease in valerate (mol % and mmol/g of DMD) with increasing 3-NOP dose was interrupted by a notable peak at 3-NOP dose MED, being significantly different from LOW and HIGH dose (*p* < 0.05), independently of the incubated diet type.

The production (mmol/g of DMD) of the branched-chain fatty acid (BCVFA) *iso*-valerate increased with increasing 3-NOP dose in a convex parabolic-shaped manner in both LC (Q: *p* = 0.048) and, as a trend, in HC (Q: *p* = 0.069) substrates (Figure 3D). However, quadratic regression coefficients were not different between LC and HC (Table 4), but i*so*-valerate was approximately 22% lower in HC when compared to LC diets (CFP: *p* < 0.05).

*Iso*-butyrate (mol %) decreased with increasing 3-NOP dose in a different manner depending on whether LC and HC diets were incubated (CFP × dose: *p* = 0.014). In HC diets, *iso*-butyrate (mol %) tended to decrease in a curvilinear-shaped manner (Q: *p* = 0.052). The 3-NOP dose levels LOW and MED did not significantly differ from PLA but noticeably declined from MED to HIGH 3-NOP dose level (*p* < 0.001). In LC treatments, *iso*-butyrate decreased more or less steadily, which led to numerically increased levels at 3-NOP dose HIGH when compared to HC treatment (Table 4).

## 4. Discussion

In the present in vitro experiment, it was hypothesised that CH_4_ production would decrease with increasing inclusion levels of 3-NOP and concentrate feeds in the incubated diet in an interactive manner.

### 4.1. 3-NOP Dosage Level

The magnitude of CH_4_ reduction was highly affected by 3-NOP inclusion level, but this occurred independently of concentrate proportion in the diet substrate. A wider range of CH_4_ inhibition was covered by the presently applied 3-NOP doses and diet substrates (Table 3) when compared to previous in vitro studies. Comparatively, Romero-Pérez et al. [10,14] incubated a forage-based substrate with 200, 500, 1000, and 2000 mg of 3-NOP/kg of feed DM in RUSITEC apparatuses. In the course of a saturation curve, they observed a high 3-NOP efficacy of reduction of 71.5, 76.0, and 84.5% at 200, 500, and 1000 mg of 3-NOP/kg of feed DM but no further CH_4_ reduction with 3-NOP dose increment from 1000 to 2000 mg of 3-NOP/kg of feed DM (84.5 and 85.6%, resp.). Interestingly, in the present study, higher CH_4_ mitigation maxima of 97 and 90% in LC and HC diets, respectively, were observed for the highest 3-NOP inclusion rate of 1200 mg/kg of feed DM applied. These different dose–response relationships may result from inherent sources of variation in the RUSITEC experiments, such as the use of different apparatuses and experimental protocols between laboratories. In the present experiment, the CH_4_ reduction (Vol.-%) increased in a linear (LC) and convex parabolic (HC) shaped manner (Figure 1A) but not as a saturation curve, as had been previously reported in vitro [10,14] and in vivo [12]. Hence, 3-NOP inhibited CH_4_ production at even lower doses (73 mg 3-NOP/kg feed DM: 12 and 10%; 160 mg 3-NOP/kg feed DM: 61 and 35% CH_4_ reduction in LC and HC diets, resp.) when compared to the 71.5% CH_4_ reduction at the minimum 3-NOP dose of 200 mg/kg of feed DM reported previously [10,23]. The differences in CH_4_ production between LOW 3-NOP dose and the PLA treatment were, however, not significant (Figure 1A). This may indicate a compensatory response by the archaeal community attempting to counterbalance the 3-NOP inhibiting effect, which was likely metabolically feasible only at the lowest 3-NOP dose. Accordingly, methanogens can reactivate MCR through internal repair systems. In fact, Duin et al. [5] concluded that CH_4_ inhibition is reversible.

Interestingly, 3-NOP dose LOW (73 mg of 3-NOP/kg of feed DM) seems to cause, in relative terms, a lower CH_4_ reduction potential under in vitro conditions (10–12%; Table 3) when compared to supplementing comparable 3-NOP dose levels to dairy cows in vivo (23% with 68 mg of 3-NOP/kg DM [24]; 26.5% and 22.5% with 60 and 80 mg of 3-NOP/kg DM, resp. [12]). Conversely, a considerably high CH_4_ reduction of more than 77.7% with 200 mg of 3-NOP/kg of feed DM can apparently only be achieved in vitro [15,23]. However, the maximum CH_4_ reduction potential seems to be limited to 40% under in vivo conditions when 3-NOP is continuously supplied at an equal dose of 200 mg/kg of feed DM to dairy cows by mixing in the compound with the TMR [12]. Thus, Melgar et al. [12] quantified the maximum CH_4_ mitigation effect to 40% at a 3-NOP dose of 100 mg/kg of feed DM without any statistical improvement in 3-NOP efficacy when supplementing higher doses of 150 and 200 mg of 3-NOP/kg of feed DM into the TMR of lactating cows. In conclusion, when compared to 3-NOP supplementation in vivo, the 3-NOP efficacy seems to be reduced at low but increased at high 3-NOP dose levels in vitro. This leads to the assumption that the dose–response relationships and 3-NOP effect mechanisms find expression in a different manner depending on whether 3-NOP is supplemented in vitro or in vivo and corresponding technical as well as rumen physiological factors affecting the mode of action of 3-NOP.

In the present experiment, the 3-NOP compound was mixed into the concentrate feed and therefore supplemented once per day as a ‘single dose’ with the feed bag into the fermenter but not as a continuous infusion. The 3-NOP compound is supposed to be water-soluble and rapidly metabolised in rumen liquid [25] and, therefore, recommended to be dosed at sufficient amounts synchronously to the MCR activity stimulating feed degradation [11]. In the present experiment, it is likely that the compound was rapidly disaggregated into 1,3-propanediol and nitrate [5] and further washed out of the vessel with the liquid outflow due to the high dilution rate of 3%/h, which could, conclusively, explain the general need for higher 3-NOP inclusion rates under conditions in vitro. In correspondence, Vyas et al. [26] observed that 3-NOP efficacy decreased 16 h after feeding when supplementing only 100 mg of 3-NOP/kg of feed DM to beef cattle, whereas a persistent CH_4_ inhibition over 24 h was achieved at higher 3-NOP inclusion levels of 200 mg/kg DM. Thus, the highest 3-NOP dose applied in the present study could have prevented the complete washing out of the 3-NOP supplement from the fermenter, which could have resulted in sufficient amounts of the feed additive remaining in the fermenter fluid for targeting archaeal MCR over the whole 24 h incubation time horizon until the next feed bag exchange. This would become even more important during the course of the incubation with regard to inactivating the MCR activity arising time-delayed from slow fermentable fibre fractions in the LC diets. Thus, rates of fermentation of NDF are significantly lower as compared to that of rapidly fermentable NFC [27]. However, the CFP × dose interaction was not significant. Moreover, RUSITEC experiments are limited to investigating the short-term gas production kinetics of fast and slow fermentable fractions between feeding bag exchange. Therefore, 3-NOP effects on 24 h fermentation kinetics should be the focus in future experiments, e.g., using the Hohenheim Gas Test, according to Menke et al. [28].

Interestingly, Duin et al. [5] found that a 100-fold increase in 3-NOP concentration is required to suppress the growth of the methanogenic Archaea *Methanomicrobium mobile* and *Methanosarcina barkeri* when compared to the required 3-NOP amounts for inhibiting the growth of the predominant species in the bovine rumen, i.e., *Methanobrevibacter ruminantium* [29]. The reasons for the different degrees of sensitivity of methanogenic species towards 3-NOP remain to be elucidated, yet 3-NOP’s effects on individual methanogenic lineages were recently observed in vivo [30,31]. However, the possibility that not all of the methanogenic species were captured by the lower 3-NOP dose could have favoured those methanogenic Archaea being less sensitive to 3-NOP, causing a shift in the methanogenic community structure to those occupying this ecological niche. As a consequence, the 3-NOP dose HIGH could have targeted a greater number and wider range of methanogenic archaeal species, causing a more comprehensive and effective blocking of those MCR amounts arising from immediate feed fermentation processes directly after feed bag exchange. This could have led to a more sustained suppressive effect on methanogenic activity until the next feed bag exchange and could further explain the high CH_4_ reduction of more than 90%. In conclusion, the 3-NOP stability and its CH_4_ inhibiting persistency in vitro should be investigated in future experiments by conducting continual 3-NOP infusion into the fermenter paralleled with frequent gas sampling from the gas bag for CH_4_ analyses between feeding events.

### 4.2. Effects of the Diet Substrate and 3-NOP on Fermentation Parameters

In the present in vitro study, the nonsignificant combination effect of 3-NOP and CFP on CH_4_ reduction contrasts findings from in vivo experiments [13,16]. This leads to the assumption that diet type per se does not contribute to synergistic effects but, rather, specific diet-induced rumen physiological factors and, more importantly, those being controlled in a RUSITEC. Thus, feeding HC diets may cause additive indirect effects on CH_4_ inhibition that are related to the increased production of propionate from H_2_-consuming fermentation pathways, passage rate (thereby limiting the time available for degradation of slowly fermentable carbohydrates), and reduced pH values (thereby inhibiting pH-sensitive methanogens) [7,32], affecting fermentation kinetics and microbial community structures [11,16,33]). As is typical for RUSITEC experiments, the fermentation conditions (e.g., particle retention time, flow rate of the (artificial) saliva, size of feed particles, motility, temperature, ratio of feed to liquid content, and liquid outflow rate) were standardised and strictly controlled in the present study. This could have equalised the abovementioned potential concentrate feed effects on fermentation characteristics and, therefore, explain the lack of synergistic effects between high CFP and 3-NOP on CH_4_ inhibition.

In the performed trial, pH values remained within the physiological range of pH-sensitive rumen bacteria and methanogenic Archaea [29]. Therefore, inhibition of rumen microorganisms due to low pH values was excluded, particularly as a high buffering capacity and controlled infusion rate of the artificial saliva were pre-set in the apparatus. The wide ratio between the liquid and solid phase in the fermenter may have prevented significant acidification solely by the diet substrate. The infusion rate of the buffer was, however, not changed with regard to the lack of effects of a comparable HC diet on rumen pH values, as previously observed in vivo [16]. In the conducted experiment, pH values were marginally lower in LC when compared to HC diets (pH: CFP: *p* = 0.019), which was related to the increased total VFA production (mmol/g of DMD) observed in LC diets (CFP: *p* = 0.012; Table 4).

End-products of microbial fermentation, i.e., VFA, act as electron acceptors, which maintain the strongly reductive rumen milieu and can, therefore, directly be linked to microbial activity [34]. Correspondingly, the *Eh* in the fluid, reflecting the redox homeostasis and electron transfer, is hypothesised to be a control of enzymatic processes in rumen microorganisms [34,35]. However, *Eh* positively correlated to total VFA (r = 0.563; N = 24; *p* = 0.004) in the present investigation, which contrasts findings of an inverse relationship [36]. From a literature review, Huang et al. [36] indicated that *Eh* increased with CFP (r = 0.497; *p* = 0.015) and negatively correlated to pH, whereby the latter was also observed in the study at hand (*Eh* vs. pH: r = −0.57; N = 24; *p* = 0.004). Hydrogen produced from microbial fermentation preserves reducing conditions in the rumen. In the present experiment, though, negative correlations between *Eh* and H_2_ concentrations in fermentation gases of 3-NOP treatments were not observed and *Eh* fluctuated inconsistently over the 3-NOP dose levels. However, interpretation should be made with caution as *Eh* was measured during feed bag exchange so that oxygen entrance, notably affecting oxidation-reduction conditions in the fermenter fluid, was unavoidable.

The present RUSITEC experiment confirmed the previous studies of Guayder et al. [23] and Romero-Peréz et al. [10] reporting H_2_ accumulation in fermentation gases and decreased molar acetate proportions with increasing 3-NOP dose and CH_4_ mitigation (H_2_ (Vol.-%): dose: *p* < 0.001 (Table 3); acetate (mol %): dose: *p* = 0.044 (Table 4)). As an H_2_-liberating fermentation process, acetate production could have been downregulated to prevent a further increase in H_2_ partial pressure in the fermenter fluid, which would be detrimental to the growth of cellulolytic bacteria. However, negative side effects of reduced NDF degradability (Table 2) or total gas production (Table 3) were not consistently observed in the present experiment. Starch fermentation in the HC diets and the 3-NOP-induced H_2_ increase (Figure 1B) were both assumed to promote a shift in fermentation balance from acetate to alternative H_2_ sinks of valerate and propionate, as previously observed under conditions in vivo [16,37]. However, contrary to these expectations, the propionate proportion (mol %) was lower in HC when compared to LC diet, whereas the opposite held true concerning valerate proportions (Table 4). However, valerate formation can be traced back not only to NFC fermentation but also to the deamination of proline. In addition, a consistent increase in propionate and valerate proportions due to the 3-NOP supply was, interestingly, observed in neither the present study nor in previous in vitro experiments [10,23], except for valerate, which was previously found to be increased with 3-NOP inclusion in vitro [23]. These observations could be explained by the unphysiological longer retention time of small-sized feed particles in a RUSITEC (fixed time of 48 h) when compared to rumen conditions in vivo. For instance, Prigge et al. [38] reported that the retention time of 3 mm particles amounts to 20 h in vivo. Martinez et al. [39] incubated a 30:70 alfalfa hay:concentrate diet in RUSITEC fermenters and observed that reducing the retention time of concentrates from 48 to 24 h and increasing the dilution rate from 3.78 to 5.42%/h increased the production and molar proportions of propionate. Furthermore, retention time depends on dry matter intake, stratification of the rumen content, and the size and density of feed particles [40]. These influencing factors were, however, standardised in the reaction vessels, which could have negatively affected the adaptation of propionate enhancers to the environmental conditions in the RUSITEC [41]. The question about a possible redirection of H_2_ spared from methanogenesis to further alternative H_2_ utilising pathways other than propionate synthesis becomes even more interesting as it can be assumed that H_2_ accumulation does not exclusively occur in fermentation gas but also in the liquid phase. Thus, in the performed experiment, the calculative amounts of the H_2_ excess from CH_4_ inhibition (assuming that 4 moles of metabolic hydrogen are spared from the inhibition of 1 mole of CH_4_) were not completely recovered in alternative H_2_ removals (H_2_ emission via fermentation gas, H_2_ incorporation into propionate and valerate), which can be deduced from the decreasing CH_4_/H_2_ ratio with increasing 3-NOP dose (Table 3). In conclusion, a rechannelling of the spared H_2_ to further alternative metabolic routes not analysed in the present experiment may have occurred. Regarding this, Guyader et al. [23] observed increased concentrations of atypical H_2_ sinks of ethanol, formate, caproate, and heptanoate in the fermenter liquid at a 200 mg inclusion rate of 3-NOP/kg of feed DM.

The convex parabolic curve of the NH_3_-N production in LC diets was inversely shaped to that of the HC diets (Figure 3A). These interrelations might be of multifactorial origin, such as reduced proteolysis or an increased microbial NH_3_-N uptake in LC fermenter fluid [42], but also with regard to the lower CP content in LC diets (Table 1). Molar proportions of *iso*-butyrate decreased with increasing 3-NOP dose, which could indicate a decreased deamination of amino acids (AA). In contrast, as reported previously [10], *iso*-valerate production increased in a quadratic manner with 3-NOP dose, which contradicts the hypothesis of a decreased AA deamination as BCVFA results from both deamination and decarboxylation of valine and leucine, respectively [43]. There are possibly different metabolic processes of these BCVFA under CH_4_ inhibition and H_2_ accumulation which need to be clarified in future (Figure 3D). Microbial uptake and release of BCVFA thus are the main determinants of BCVFA concentrations and microbial protein synthesis is regarded as a H_2_ sink apart from methanogenesis [43,44].

## 5. Conclusions

The hypothesis of synergistic effects between 3-NOP and increased CFP on CH_4_ inhibition was rejected for the applied in vitro conditions. The present RUSITEC experiment evidenced that 3-NOP effectively inhibited methanogenesis in a dose-dependent manner irrespective of CFP in the incubated diet. Negative side effects on nutrient degradability and, correspondingly, total VFA and gas production were not consistently observed for 3-NOP or CFP. However, 3-NOP dose increment was paralleled by H_2_ gas accumulation, whereas alternative H_2_ sinks of propionate and valerate remained unaffected. Increasing 3-NOP dosage decreased H_2_-liberating acetate formation, whereas butyrate proportion remained unchanged. Conclusions from in vitro experiments cannot be fully transferred to the rumen environment in vivo. This study and others suggest that extrapolating findings from dose-dependent dynamics of the 3-NOP efficacy under conditions in vitro should be treated with caution for planning 3-NOP application in vivo. The present research should be broadened by focusing on potential changes in microbial community structures when 3-NOP is supplemented to different dietary concentrate:forage ratios.

## Figures and Tables

**Table 1:** Ingredients and chemical composition of the experimental diets.

	Experimental Diet ^†^	
Item	LC	HC
Ingredients (g/kg of diet DM ^§^)		
Maize silage	495	286
Grass silage	212	122
Rapeseed meal	44.4	90.1
Soybean meal	37.2	74.7
Wheat	97	195.5
Dried sugar beet pulp	85	172
Soybean oil	4.5	9.2
Calcium carbonate	7	14.2
Urea	2.9	6.1
Vitamin/Mineral premix ^+^	15	30.2
Chemical analysis of the ration		
DM (g/kg)	908	897
Nutrient (g/kg of DM)		
Organic matter	923	938
Crude protein	131	171
Ether extract	33	33
aNDFom ^¶^	382	308
ADFom ^#^	217	178
Starch	257	284

^†^ Experimental diets with low- (LC) and high- (HC) concentrate feed proportion supplied at four doses of 0, 0.073, 0.16, and 1.2 mg of the active 3-nitrooxypropanol substance/g feed DM. ^§^ DM, dry matter. ^+^ Ingredients according to the manufacturer’s specifications: minerals (g/kg of premix): Ca, 140; Na, 120; P, 70; Mg, 40; Zn, 6; Mn, 5.4; Cu, 1; I, 0.1; Se, 0.04; Co, 0.025; vitamins (IU/kg of premix): A, 1,000,000; D3, 100,000; E, 2235. ^¶^ aNDFom; α-amylase treated neutral detergent fiber expressed without residual ash; ^#^ ADFom; acid detergent fibre expressed without residual ash.

**Table 2:** Effects of 3-nitrooxypropanol (3-NOP) dosage levels (PLA: 0, LOW: 73, MED: 160, and HIGH: 1200 mg of 3-NOP/kg of feed DM) and low- (LC) or high- (HC) concentrate feed proportion in the diet (CFP) on dry matter degradation (DMD), apparent organic matter degradation (OMAD), and neutral-detergent fibre degradation (NDFD) (g/kg DM).

Item	Treatments ^†^					SEM ^§^	*p*-Values ^+^						
	CFP	PLA	LOW	MED	HIGH		CFP	Dose	CFP × Dose	L	Q	*ß*_L_ > *F*	*ß*_Q_ > *F*
DMD	LC	640	718	638	596	26	0.640	**0.041**	0.335	0.464	0.229	0.364	0.216
	HC	655	687	627	658					0.703	0.743		
OMAD	LC	643	723	642	601	26	0.654	0.052	0.254	0.395	0.170	0.275	0.136
	HC	658	687	628	669					0.621	0.620		
NDFD	LC	358	505	362	293	49	0.091	0.246	0.145	0.346	0.149	0.349	0.162
	HC	310	319	288	350					0.699	0.585		

^†^ Values presented as LS means. ^§^ SEM, standard error of the means. ^+^ Effects of CFP, 3-NOP dose, and interactions between them; L, Q, *p*-values for linear and quadratic effects of 3-NOP; *ß*_L_ > *F*, *ß*_Q_ > *F*, probability under *H*_0_ that an F-distributed random variable exceeds observed *F*, for the difference in the linear and quadratic regression coefficients between LC and HC. Significant values (*p* ≤ 0.05 are highlighted in bold).

**Table 3:** Effects of 3-nitrooxypropanol (3-NOP) dosage levels (PLA: 0, LOW: 73, MED: 160, and HIGH: 1200 mg of 3-NOP/kg of feed DM) and low- (LC) or high- (HC) concentrate feed proportion in the diet (CFP) on fermentation gas production and composition.

Item	Treatments ^†^					SEM ^§^	*p*-Values ^+^						
	CFP	PLA	LOW	MED	HIGH		CFP	Dose	CFP × Dose	L	Q	*ß*_L_ > *F*	*ß*_Q_ > *F*
Total GP ^¶^ (mL/d)	LC	1902	1904	1664	1818	18	0.506	0.600	0.978	0.411	0.479	0.823	0.732
	HC	1844	1797	1648	1654					0.543	0.705		
**Gas production (mL/g of DMD** ^#^ **)**													
Total GP	LC	1091	977	938	1152	110	0.494	0.711	0.698	0.181	0.121	0.423	0.272
	HC	1042	970	982	944					0.703	0.825		
CH_4_	LC	15.8	12.2	5.2	0.5	1.2	0.419	**<0.001**	0.241	**0.001**	0.145	**0.028**	**0.045**
	HC	14.5	12.1	8.7	1.1					0.133	0.407		
CO_2_	LC	100.2	94.2	92.7	98.4	13.8	0.538	0.962	0.981	0.642	0.637	0.835	0.745
	HC	93.4	94.1	86.6	86.8					0.812	0.907		
H_2_	LC	0.44	2.11	6.03	11.89	1.92	0.579	**0.001**	0.623	0.290	0.766	0.595	0.859
	HC	1.42	3.21	3.80	8.96					0.749	0.584		
**Gas composition (Vol.-%)**													
CH_4_	LC	5.8	5.1	2.2	0.2	0.3	0.138	**<0.001**	0.094	**0.002**	0.566	0.082	0.124
	HC	5.5	5.0	3.6	0.5					0.241	0.119		
CO_2_	LC	36.6	38.5	39.6	33.5	1.9	0.567	0.430	0.302	0.146	0.066	0.173	0.086
	HC	35.6	37.8	35.0	36.6					0.979	0.959		
H_2_	LC	0.2	0.9	2.6	3.9	0.5	0.755	**<0.001**	0.458	**0.046**	0.579	0.233	0.307
	HC	0.5	1.2	1.5	3.7					0.699	0.368		
CH_4_/CO_2_	LC	0.157	0.131	0.055	0.004	0.008	0.024	<0.001	**0.026**	**<0.001**	0.220	**0.035**	**0.048**
	HC	0.155	0.133	0.102	0.014					0.177	0.082		
CO_2_/CH_4_	LC	6.4	7.8	18.9	915.6	193.2	0.185	**0.036**	0.179	0.398	0.066	0.592	0.238
	HC	6.5	7.6	10.0	163.6					0.887	0.741		
CH_4_/H_2_	LC	39.45	5.84	0.86	0.04	2.65	0.002	<0.001	**0.001**	**<0.001**	**0.001**	**0.001**	**0.002**
	HC	10.53	4.32	3.00	0.22					0.183	0.422		

^†^ Values presented as LS means. ^§^ SEM, standard error of the means. ^+^ Effects of CFP, 3-NOP dose, and interactions between them; L, Q, *p*-values for linear and quadratic effects of 3-NOP; *ß*_L_ > *F*, *ß*_Q_ > *F*, probability under *H*_0_ that an F-distributed random variable exceeds observed *F*, for the difference in the linear and quadratic regression coefficients between LC and HC. ^¶^ GP, gas production. ^#^ DMD, dry matter degradation. Significant values (*p* ≤ 0.05 are highlighted in bold).

**Table 4:** Effects of 3-nitrooxypropanol (3-NOP) dosage levels (PLA: 0, LOW: 73, MED: 160, and HIGH: 1200 mg of 3-NOP/kg of feed DM) and low- (LC) or high- (HC) concentrate feed proportion in the diet (CFP) on fermentation characteristics and production of volatile fatty acids (VFA).

Item	Treatments ^†^					SEM ^§^	*p*-Values ^+^						
	CFP	PLA	LOW	MED	HIGH		CFP	Dose	CFP × Dose	L	Q	*ß*_L_ > *F*	*ß*_Q_ > *F*
pH	LC	6.39	6.29	6.44	6.42	0.05	**0.019**	0.552	0.083	0.912	0.830	0.880	0.495
	HC	6.49	6.51	6.48	6.40					0.753	0.470		
*Eh* (mV) ^¶^	LC	−279	−244	−302	−268	12	0.066	**0.010**	0.517	0.623	0.645	0.100	0.178
	HC	−308	−271	−299	−281					0.621	0.745		
Effluent (mL/d)	LC	656	588	650	655	25	0.950	0.789	0.065	0.442	0.328	0.433	0.361
	HC	622	666	625	642					0.731	0.752		
NH_3_-N (mg/L)	LC	157	165	136	142	8	**0.009**	**0.006**	0.499	0.344	0.611	0.187	0.125
	HC	214	235	207	191					0.608	0.228		
NH_3_-N(mg/g DMD ^#^)	LC	14.9	12.4	12.5	14.7	1.1	**<0.001**	0.861	0.081	0.065	**0.049**	**0.040**	**0.020**
	HC	18.3	20.9	19.1	17.7					0.260	0.151		
Total VFA (mmol/L)	LC	76.3	83.4	78.0	71.1	5.1	0.250	0.849	0.203	0.466	0.287	0.547	0.229
	HC	69.0	70.6	71.3	80.5					0.955	0.587		
**Fermentation pattern (mol % of VFA)**													
Acetate	LC	53.4	53.1	49.4	51.0	1.14	0.548	**0.044**	0.705	0.097	0.193	0.180	0.267
	HC	51.4	52.3	49.8	50.7					0.594	0.730		
Propionate	LC	17.9	18.4	18.1	13.8	0.73	0.326	0.012	**0.029**	0.197	**0.017**	0.683	0.135
	HC	16.2	15.9	17.2	16.0					0.387	0.385		
Butyrate	LC	13.6	13.5	15.5	15.3	1.13	0.190	0.864	0.281	0.414	0.629	0.495	0.988
	HC	16.7	16.7	16.1	14.6					0.964	0.641		
*Iso*-butyrate	LC	0.91	0.82	0.83	0.79	0.03	0.003	<0.001	**0.014**	0.134	0.371	0.170	**0.034**
	HC	0.98	1.00	0.92	0.74					0.821	0.052		
Valerate	LC	4.9	4.3	5.8	4.5	0.39	0.122	**0.005**	0.255	0.238	0.209	0.772	0.872
	HC	6.2	5.9	6.5	4.8					0.396	0.152		
*Iso*-valerate	LC	9.3	9.8	10.4	14.7	0.83	**0.044**	**<0.001**	0.893	0.828	0.102	0.797	0.867
	HC	8.6	7.9	9.4	13.1					0.569	0.066		
C_2_/C_3_ ratio ^$^	LC	3.03	2.92	2.77	3.80	0.18	0.832	0.011	**0.032**	0.068	**0.008**	0.414	0.090
	HC	3.23	3.36	2.91	3.19					0.335	0.392		
**VFA production (mmol/g of DMD)**													
Total VFA	LC	7.18	6.86	7.11	7.36	0.264	**0.012**	**0.050**	0.409	0.569	0.416	0.468	0.997
	HC	6.22	6.26	6.56	7.32					0.927	0.418		
Acetate	LC	3.85	3.64	3.52	3.73	0.131	**0.004**	0.105	0.143	0.079	0.090	0.194	0.583
	HC	3.20	3.27	3.27	3.73					0.799	0.266		
Propionate	LC	1.30	1.27	1.29	1.02	0.073	0.022	0.531	0.031	0.566	0.191	0.950	0.155
	HC	1.02	1.00	1.14	1.19					0.604	0.997		
Butyrate	LC	0.96	0.93	1.09	1.13	0.087	0.777	0.551	0.640	0.554	0.931	0.645	0.994
	HC	1.04	1.05	1.05	1.05					0.898	0.925		
*Iso*-butyrate	LC	0.065	0.060	0.059	0.058	0.003	0.421	0.134	0.605	0.198	0.384	0.149	0.126
	HC	0.060	0.062	0.060	0.055					0.725	0.366		
Valerate	LC	0.34	0.30	0.41	0.33	0.029	0.256	**0.035**	0.469	0.245	0.265	0.848	0.944
	HC	0.39	0.38	0.42	0.35					0.346	0.232		
*Iso*-valerate	LC	0.68	0.66	0.74	1.09	0.071	**0.012**	**<0.001**	0.991	0.539	**0.048**	0.858	0.894
	HC	0.52	0.49	0.62	0.95					0.716	0.069		

^†^ Values presented as LS means. ^§^ SEM, standard error of the means. ^+^ Effects of CFP, 3-NOP dose, and interactions between them; L, Q, *p*-values for linear and quadratic effects of 3-NOP; *ß*_L_ > *F*, *ß*_Q_ > *F*, probability under *H*_0_ that an F-distributed random variable exceeds observed *F*, for the difference in the linear and quadratic regression coefficients between LC and HC. ^¶^ *Eh*, redox potential. ^#^ DMD, dry matter degradation. ^$^ C_2_/C_3_ ratio, acetate/propionate ratio. Significant values (*p* ≤ 0.05 are highlighted in bold).

## Data Availability

The data presented in this study are available on request from the corresponding author. The data are not publicly available due to legal issues.
